# Serious Invasive Saffold Virus Infections in Children, 2009

**DOI:** 10.3201/eid1801.110725

**Published:** 2012-01

**Authors:** Alex Christian Yde Nielsen, Blenda Böttiger, Jytte Banner, Thomas Hoffmann, Lars Peter Nielsen

**Affiliations:** University of Southern Denmark, Odense, Denmark (A.C.Y. Nielsen);; Statens Serum Institut, Copenhagen, Denmark (A.C.Y. Nielsen, B. Böttiger, L.P. Nielsen);; University of Aarhus, Aarhus, Denmark (J. Banner);; Hvidovre Hospital, Copenhagen (T. Hoffmann)

**Keywords:** Saffold virus, cardiovirus, viral encephalitis, encephalitis, cerebrospinal fluid, picornavirus infections, fatal outcome, viruses, Denmark, children

## Abstract

The first human virus in the genus *Cardiovirus* was described in 2007 and named Saffold virus (SAFV). Cardioviruses can cause severe infections of the myocardium and central nervous system in animals, but SAFV has not yet been convincingly associated with disease in humans. To study a possible association between SAFV and infections in the human central nervous system, we designed a real-time PCR for SAFV and tested cerebrospinal fluid (CSF) samples from children <4 years of age. SAFV was detected in 2 children: in the CSF and a fecal sample from 1 child with monosymptomatic ataxia caused by cerebellitis; and in the CSF, blood, and myocardium of another child who died suddenly with no history of illness. Virus from each child was sequenced and shown to be SAFV type 2. These findings demonstrate that SAFV can cause serious invasive infection in children.

## Abstract 2

This virus might have caused previously unexplained cerebral infections and deaths in children.

Molecular biology has revolutionized the diagnostics of infectious diseases through the introduction of more sensitive and specific diagnostic tests. Despite these advances, the etiologic agents of many apparent infections are still unidentified. For example, the etiologic agent is unknown for many cases of apparent pneumonia (*1*); in a study conducted in California, USA, despite extensive testing and evaluation, an underlying cause of encephalitis was unidentified for 207 (62%) of 334 patients (*2*).

During the past few years, intensive searches for new viruses, using conventional virologic methods and metagenomics, have resulted in the discovery of several new viruses. During the past decade, the family *Picornaviridae* has grown as the number of recognized genera has increased from 6 to 12 (*3*,*4*); the numbers of species, types, and subtypes have increased even more. However, only viruses from 3 genera (*Enterovirus*, *Hepatovirus*, and *Parechovirus*) have been firmly established as being capable of causing clinically significant disease in humans. Viruses from other genera (*Cardiovirus*, *Cosavirus*, and *Kobuvirus*) have so far been detected only in noninvasively collected human sample material such as fecal and respiratory samples, and their clinical significance remains to be fully elucidated. (Invasively collected sample material is that from tissues considered sterile, i.e., devoid of microorganisms.)

The phylogenetic relationships of human picornaviruses are shown in Figure 1. Most picornaviruses that are pathogenic to humans are ubiquitous viruses capable of causing a variety of diseases, from monosymptomatic febrile infection to severe infection in the central nervous system and myocardium. However, most infections with these viruses are asymptomatic (*5*).

**Figure 1:**
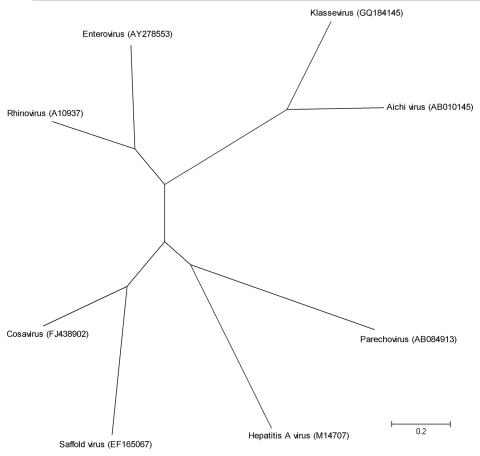
Phylogenetic tree based on full-genome sequences of all known human picornavirus species, represented with 1 virus strain each. The tree was constructed by the neighbor-joining method by using MEGA4 software (www.megasoftware.net). Scale bar indicates nucleotide substitutions per site.

Saffold virus (SAFV) was discovered by Jones et al. in 2007 by sequence-independent genomic amplification of virus isolated from a fecal sample (*6*). The sample had been obtained in 1981 from an 8-month-old child with fever of unknown origin. The genetic sequence of the virus indicated that the virus belonged to the species *Theilovirus* of the genus *Cardiovirus*, which contains 3 other members: Theiler’s murine encephalomyelitis virus (TMEV), Vilyuisk human encephalomyelitis virus (VHEV), and Thera virus. In mice, TMEV is capable of causing infection in the central nervous system, and some variants of this virus cause a persistent infection and even multiple sclerosis­­–like disease (*7*). VHEV was isolated in the 1950s from cerebrospinal fluid (CSF) from a patient with Vilyuisk encephalomyelitis, a progressive neurologic disorder that occurs in indigenous populations of an isolated part of eastern Siberia (*8*). However, the correlation between VHEV and Vilyuisk encephalomyelitis is still uncertain because VHEV has been isolated by multiple passages in mice and thus may represent a highly divergent strain of TMEV. Thera virus (previously named Theiler-like rat virus) has been isolated from rats, but the clinical significance of infections with this virus is unknown (*9**,**10*). The genus *Cardiovirus* also contains a second species called encephalomyocarditis virus. Only 1 serotype is known, and it is capable of causing encephalitis and myocarditis in various animals (*11**,**12*).

Since the discovery of SAFV, several articles have provided insight into its epidemiologic and, to a minor degree, clinical significance. Saffold viruses are distributed worldwide (*6**,**13**–**19*), and 2 serologic studies have demonstrated that infection occurs early in life (*14**,**20*). However, finding an association with human disease has thus far been elusive. Most studies (*13**,**15**,**17**,**20**–**22*) have tried to associate SAFV with gastroenteritis, but no convincing results have been produced. A few studies (*16**,**18**,**21**,**23*) analyzed the clinical significance of SAFV virus in the respiratory system, but no substantial association between the virus and respiratory symptoms or disease has been made. Only 1 study (*21*) reports having tested invasively collected sample material (CSF samples), but no findings were positive.

To investigate the possible invasive potential of SAFV in humans, we developed a diagnostic PCR and tested CSF samples from a group of children. SAFV was detected in 2 of these children.

## Materials and Methods

We tested previously submitted CSF samples for SAFV, reviewed the patients’ medical records, and sequenced the viruses isolated. The study was approved by the local ethics committee, De Videnskabsetiske Komiteer for Region Hovedstaden, Denmark, protocol no. H-2–2010–019.

### CSF Specimens

We tested 332 consecutively submitted CSF samples from 319 patients <4 years of age from Denmark that had been submitted to Statens Serum Institut, Copenhagen, from January 2006 through December 2009 for viral diagnostic testing. We tested for the following viruses: herpes simplex virus types 1 and 2 (228 samples), varicella zoster virus (228 samples), human enterovirus (261 samples), and human parechovirus (88 samples since November 2008). For all samples, initial test results were negative.

### Fecal Samples

We selected fecal samples from 479 children <5 years of age with gastroenteritis that had been submitted for viral diagnostic testing from September 2009 through February 2010 and tested them for SAFV. Nucleic acid extracted from these samples was combined into 48 pools, with 9 or 10 samples per pool. Samples from pools with positive results were identified, and new extractions from these pools were tested individually. However, enough sample material for new extractions was available for only about half of the samples.

### Nucleic Acid Purification

Nucleic acids were extracted from 200 µL of CSF or blood (from SAFV-positive patients) by using the QIAamp DNA Blood Mini Kit (QIAGEN, Hilden, Germany) and semiautomatic extraction on the QIAcube instrument (QIAGEN). Nucleic acid was extracted from 200-µL fecal suspension (10% in phosphate-buffered saline) by using the Total Nucleic Acid Isolation Kit (Roche Diagnostics GmbH, Mannheim, Germany) on the MagnaPure LC instrument (Roche Diagnostics GmbH).

### Nucleic Acid Amplification and Detection

Five microliters of extracted material was used per reverse transcription PCR (total volume 25 µL) by using the OneStep RT-PCR Kit (QIAGEN). The reaction mixtures contained 1 µmol/L of each primer and 0.2 µmol/L of probe.

Design of the primers and probe was based on an alignment of all available SAFV sequences in GenBank (www.ncbi.nlm.nih.gov/genbank) in July 2010 by using ClustalW (www.clustal.org) and Primer3 (http://frodo.wi.mit.edu/primer3) software. The primers and probe are selective for a highly conserved part of the 5′ untranslated region (Table). The Strategene Mx3005P real-time thermocycler instrument (Agilent Technologies A/S, Horsholm, Denmark) was used for amplification and detection with the following settings: 50°C for 20 min, 95°C for 15 min, followed by 45 cycles of 95°C for 15 s and 55°C for 1 min.

### Genotyping by PCR and Sequencing

Genotyping was conducted by nested PCR and sequencing of parts of the viral protein (VP) 1 and VP2 regions of the capsid gene by using primers listed in the Table. The inner VP1 and VP2 primers amplified DNA fragments of ≈599 and 577 bp, respectively. PCR products were purified by using the High Pure PCR Purification Kit (Roche Diagnostics GmbH) before sequencing, which was performed by using the inner PCR primers on an ABI automated sequencer and BigDye version 1.1 chemistry (both from Applied Biosystems, Darmstadt, Germany). Sequences were aligned, and phylogenetic analysis with known reference sequences was performed by using MEGA4 software (www.megasoftware.net). The sequences have been submitted to GenBank under accession nos. JF693612–23.

## Results

SAFV was detected in CSF from 2 of the 319 children. Additional sample material from these 2 children was subsequently obtained and tested. From child 1, blood and CSF collected at the same time and a fecal sample collected 2 weeks later were tested; only test results for the fecal sample were positive for SAFV. From child 2, a postmortem blood sample and a myocardial biopsy sample were tested; test results for each sample were positive for SAFV.

### Child 1

Child 1 was a 16-month-old, previously healthy boy who became ill in May 2009. The boy had a fever 6 days before hospital admission, followed 1 day later by sudden onset of monosymptomatic ataxia, with no fever. The ataxia fluctuated from causing an insecure gait to walking into things and falling. The patient also had intermittent difficulty controlling his arms when trying to eat. No history of recent travel was reported. The boy was in otherwise good health; he had no abnormal psychological symptoms and retained a normal degree of consciousness throughout the acute phase of the disease. Differential diagnoses at hospital admission were intracranial tumor or viral cerebellitis. The boy’s 4-year-old sister remained healthy.

Laboratory test results are listed in Table A1. At hospital admission, CSF values (leukocyte count, protein level, and glucose level) were within reference ranges, and no microorganisms were detected. A magnetic resonance imaging scan of the brain showed a small venous anomaly in the left frontal lobe but no tumor, hemorrhage, or inflammation. A fecal sample collected 2 weeks later yielded positive test results for parechovirus type 3 and negative results for enterovirus and adenovirus. Parechovirus was not found in the CSF or blood.

During the next 2 months, the ataxia remitted completely without sequelae. The diagnosis at this time was viral encephalitis, possibly caused by parechovirus type 3.

### Child 2

Child 2 was a 27-month-old, previously healthy girl who was found dead in her bed in August 2009; she had no known history of disease or symptoms. During necropsy, signs of cerebral herniation were detected. A small vascular malformation surrounded by edema was found in the brain. No signs of encephalitis or hemolytic uremic syndrome were found.

The following sample materials were collected: CSF, blood, feces, myocardium, pericardial aspirate, lung tissue, and respiratory secretions (Table A1). In the CSF, mononuclear pleocytosis was noted. Results of routine bacteriologic culture found coagulase-negative staphylococci in the CSF, a few nonhemolytic streptococci in the lung tissue, and a few nonhemolytic streptococci and a few *Staphylococcus aureus* organisms in the pericardial aspirate. Verotoxin-producing *Escherichia coli* was cultured from the fecal sample. The conclusion of the autopsy and laboratory findings was that cerebral herniation was the immediate cause of death.

**Figure 2:**
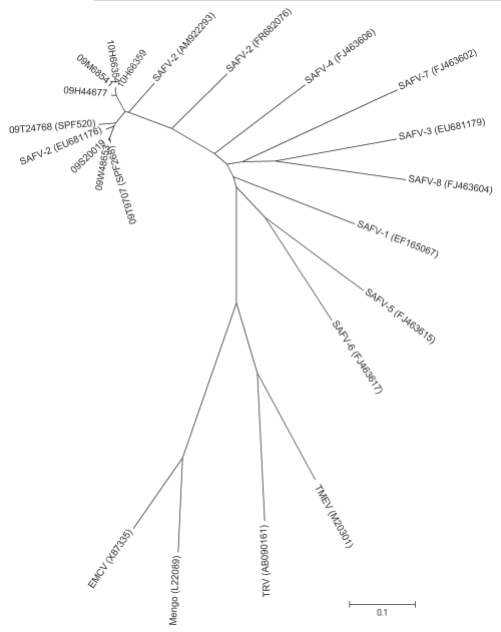
Phylogenetic tree showing partial viral protein 1 capsid sequences (588 nt) from the Saffold virus strains identified from 2 children from Denmark, 2009, and representative strains from other cardioviruses. The tree was constructed by the neighbor-joining method by using MEGA4 software (www.megasoftware.net). Scale bar indicates nucleotide substitutions per site. SAFV, Saffold virus; TMEV, Theiler’s murine encephalomyelitis virus; TRV, Thera virus; EMCV, encephalomyocarditis virus.

Virus from each of the 2 children was characterized by sequencing part of the VP1 region of the capsid gene, and the sequences were compared with those of other SAFVs detected in fecal samples from the patients with gastroenteritis (Figure 2). Of the 48 fecal sample pools, 10 were positive for SAFV by PCR. From these pools, 6 individual samples were available for further testing; the VP2 capsid region was successfully sequenced for 4 of these 6 samples, and they were all SAFV type 2. Later, an SAFV type 2–specific VP1 PCR was designed, which provided VP1 sequences from all 6 fecal samples and samples from the 2 children reported here: the fecal sample from child 1 and the blood sample from child 2. The phylogenetic analysis (Figure 2) showed that all viruses were SAFV type 2 and that the sequences arranged in 2 clusters with 8% nt differences and 5% aa differences between the clusters.

## Discussion

Several novel viruses have recently been discovered by using microarrays or mass-sequencing methods (*6**,**21*). Almost all of these viruses have been found in noninvasively collected patient materials, making the correlation to clinical disease difficult to establish. For the picornavirus group, this issue is further complicated by knowledge that this virus family can cause a wide variety of diseases with a high proportion of nonspecific symptoms or asymptomatic infections (*5*).

We report 2 cases of invasive infection with SAFV type 2 in children. For each child, SAFV was detected in at least 1 compartment other than the central nervous system. This finding strengthens the evidence of an acute infection as the cause of clinical disease. In child 1, SAFV virus was found in the CSF and a fecal sample. In child 2, findings were even more convincing because SAFV was found in 3 invasively collected samples: CSF, myocardium, and blood. No other credible cause of infection was found for either of the 2 children. In child 1, the only other positive finding was parechovirus type 3 in a fecal sample taken 2 weeks after onset of disease. Parechovirus was not found in the CSF and therefore seems unlikely as the cause of the acute symptoms. From child 2, several types of bacteria were identified, but these seem unlikely to be the cause of death because culture of postmortem samples often grows commensal organisms. The bacteria from this child were not found consistently in the tissue samples, thus making systemic bacterial infection less likely. Verotoxin-producing *E. coli* also seems an unlikely cause of death because no diarrhea or signs of hemolytic uremic syndrome were present before the child’s death or at autopsy.

At autopsy of child 2, a small vascular malformation surrounded by edema was found. This edema could be the cause of the herniation. However, the edema might also have been caused by cerebral infection or septicemia. This possibility is supported by the finding of mononuclear cells in the spinal fluid and the direct virus identification in the CSF and blood. The exact cause of death and whether there is a connection between the infection and the changes surrounding the malformation are unclear. However, our investigations show that before her death, the child’s blood contained SAFV.

Child 1 had monosymptomatic ataxia preceded by 1 day of fever. The symptoms receded over the next few months, and the patient recovered fully. Child 2 died without any preceding symptoms or any known predisposing factors; this clinical picture is sometimes found for patients with enteroviral infections (*24*).

The finding of SAFV in invasively collected samples from the 2 children described here fits well with the knowledge about the picornavirus group. SAFV probably behaves similarly to the viruses in the enterovirus group, producing mainly asymptomatic infections but also producing nonspecific symptoms in other patients and severe disease in a few patients. Enteroviruses are known to cause many more or less organ-specific diseases such central nervous system infection (meningitis, encephalitis, and myelitis), myocarditis, enanthema, exanthema, and septicemia. These 2 cases fit well within the expected range of diseases attributed to picornaviruses, but further studies are needed to determine the exact correlation of SAFV to disease in humans.

In a previous study, Chiu et al. looked for SAFV in 400 CSF samples but found none (*21*). Lack of detection could be explained by different assay sensitivities or by the selection of samples tested. In their study, patient selection was based on neurologic disease and patient ages were not reported.

Our study has also shown that Saffold virus type 2 circulated in Denmark in 2009, the same year that the children reported here became ill. Other studies have shown type 2 to be a common type of SAFV and to be circulating worldwide (*13**,**15**,**16**,**18**,**20**,**21**,**23*). Because SAFVs are single-stranded RNA viruses, a nucleotide variation of 8% among the restricted number of samples in this study is expected.

In conclusion, we have established SAFV virus as a cause of invasive infection and a highly probable cause of severe disease in children. More studies are needed to further illuminate the role of SAFV as a human pathogen.

## Appendix

**Table A1:** Laboratory results for 2 children with Saffold virus infection, Denmark, 2009

Analysis	Child 1†				Child 2‡						
	Spinal fluid	Blood	Feces		Spinal fluid	Blood	Myocardial tissue	Pericardial fluid	Lung tissue	Respiratory samples	Feces
Biochemistry											
Albumin	0.09 g/L	–	–		–	–	–	–	–	–	–
C-reactive protein	–	<6 mg/L	–		–	–	–	–	–	–	–
Creatinine	–	41 µmol/L	–		–	–	–	–	–	–	–
Glucose	3.6 mmol/L	–	–		–	–	–	–	–	–	–
Hemoglobin	–	7.8 mmol/L	–		–	–	–	–	–	–	–
IgG	–	5.8 g/L	–		–	–	–	–	–	–	–
Thrombocytes	–	301 × 10^9^/L	–		–	–	–	–	–	–	–
Protein	0.14 g/L	–	–		–	–	–	–	–	–	–
Sodium	–	140 mmol/L	–		–	–	–	–	–	–	–
Erythrocytes	0 × 10^6^/L	–	–		–	–	–	–	–	–	–
Leukocytes	8 × 10^6^/L	8.4 × 10^9^/L	–		–	–	–	–	–	–	–
Culture: bacteria	Negative	–	–		CNS	–	–	A few NHS	A few NHS and *Staphlylococcus aureus*	–	VTEC
ELISA											
* Borrelia burgdorferi*	–	Negative IgM and IgG	–		–	–	–	–	–	–	–
Endomysium antibodies	–	–	–		–	Negative	–	–	–	–	–
Epstein Barr virus	–	EBNA IgG negative	–		–	–	–	–	–	–	–
*Haemophilus influenzae* type b	–	–	–		–	0.62 µg/mL	–	–	–	–	–
* Legionella pneumophilae*	–	Negative IgM and IgG	–		–	–	–	–	–	–	–
* Mycoplasma pneumoniae*	–	Negative IgM and IgG	–		–	–	–	–	–	–	–
Intrathecal synthesis											
* Borrelia burgdorferi*	Negative	–	–		–	–	–	–	–	–	–
Herpes simplex virus	Negative	–	–		Negative	–	–	–	–	–	–
Ig	Negative	–	–		–	–	–	–	–	–	–
Varicella zoster virus	Negative	–	–		Negative	–	–	–	–	–	–
PCR											
Adenovirus	Negative	–	Negative		–	Negative	Negative	–	–	Negative	–
* Chlamydophila pneumoniae*	–	–	–		Negative	–	–	–	–	–	–
* Chlamydophila psittaci*	–	–	–		Negative	–	–	–	–	–	–
Coronavirus	–	–	–		–	–	–	–	–	Negative	–
Enterovirus	Negative	–	Negative		Negative	Negative	Negative	–	–	–	Negative
Epstein Barr virus	Negative	Negative	–		–	Negative	–	–	–	–	–
Herpes simplex virus	Negative	Negative	–		Negative	–	–	–	–	–	–
Influenza viruses	–	–	–		–	–	–	–	–	Negative	–
* Legionella pneumophilae*	–	–	–		Negative	–	–	–	–	–	–
Metapneumovirus	–	–	–		–	–	–	–	–	Negative	–
* Mycoplasma pneumoniae*	–	–	–		Negative	–	–	–	–	–	–
* Neisseria meningitidis*	–	–	–		Negative	–	–	–	–	–	–
Parainfluenza viruses	–	–	–		–	–	–	–	–	Negative	–
Parechovirus	Negative	–	Positive		Negative	Negative	Negative	–	–	–	Negative
Parotitis virus	Negative	Negative	–		–	–	–	–	–	–	–
Parvovirus	–	–	–		–	Negative	Negative	–	–	–	–
Respiratory syncytial virus	–	–	–		–	–	–	–	–	Negative	–
Rhinovirus	–	–	–		–	–	–	–	–	Negative	–
* Streptococcus pneumoniae*	–	–	–		Negative	–	–	–	–	–	–
Varicella zoster virus	Negative	Negative	–		Negative	–	–	–	–	–	–

*–, not tested or not relevant; CNS, coagulase-negative staphylococci; NHS, nonhemolytic streptococci; VTEC, verotoxin-producing *Escherichia coli*. †Previously healthy16-month-old boy who became ill in May 2009. ‡Previously healthy 27-month-old girl who was found dead in her bed in August 2009.

**Table:** Primers and probe used for diagnostic PCR and primers used to sequence Saffold virus, 2009*

Assay, primer	Type	Sequence, 5′ → 3′
Diagnostic RT-PCR		
Saffold F	Forward primer	CTA WCA TGC CTC CCC GAT T
Saffold R	Reverse primer	GYT TAG ACC GGG GGA ACC
Saffold Probe	Probe	TTT CTG CCC TGC TGG GCG G
VP2 typing		
SAFV VP2 OF	Outer forward primer	GAR ATG ACY AAY CTB TCW GAY AGA GT
SAFV VP2 OR	Outer reverse primer	CCR TTR AAN ACS GGY TTN AC
SAFV VP2 IF	Inner forward primer	CGG CYA YAA ACA CKC AAT C
SAFV VP2 IR	Inner reverse primer	TTD GCR TGY TGN GTC CA
VPI typing, type 2 specific		
SAFV-2 VP1 OF1	Outer forward primer 1	GCA GAA AAA GGA AAG GTT GC
SAFV-2 VP1 OF2	Outer forward primer 2	GCA GAA AAA GGC AAA GTT GC
SAFV-2 VP1 OR	Outer reverse primer	TCT TGG RCA AAA CAC TCT CA
SAFV-2 VP1 IF	Inner forward primer	CYA TAG CTC TTC CTG AAA AYC A
SAFV-2 VP1 IR	Inner reverse primer	TGR ACC GAA AAY CTG TCT GC

*RT-PCR, real-time reverse transcription PCR; SAFV, Saffold virus: VP, viral protein.

## References

[1] Mandell LA, Wunderink RG, Anzueto A, Bartlett JG, Campbell GD, Dean NC, Infectious Diseases Society of America/American Thoracic Society consensus guidelines on the management of community-acquired pneumonia in adults. Clin Infect Dis. 2007;44(Suppl 2):S27–72. 10.1086/511159 17278083 PMC7107997

[2] Glaser CA, Gilliam S, Schnurr D, Forghani B, Honarmand S, Khetsuriani N, In search of encephalitis etiologies: diagnostic challenges in the California Encephalitis Project, 1998–2000. Clin Infect Dis. 2003;36:731–42. 10.1086/367841 12627357

[3] King AM, Brown F, Christian P, Hovi T, Hyypia T, Knowles NJ, Picornaviridae. In: van Regenmortel MH, Fauquet CM, Bishop DH, Carsten EB, Estes MK, Lemon SM, et al., editors. Virus taxonomy. Seventh report of the International Committee for the Taxonomy of Viruses. New York: Academic Press; 2000. p. 657–73.

[4] International Committee for the Taxonomy of Viruses. ICTV master species list 2009–version 9 [updated 2009 Oct 21; cited 2011 Mar 2]. http://talk.ictvonline.org/files/ictv_documents/m/msl/1231.aspx

[5] Dagan R, Menegus MA. Nonpolio enteroviruses and the febrile infant. In: Rotbart HA, editor. Human enterovirus infections. Washington: ASM Press; 1995. p. 239–54.

[6] Jones MS, Lukashov VV, Ganac RD, Schnurr DP. Discovery of a novel human picornavirus in a stool sample from a pediatric patient presenting with fever of unknown origin. J Clin Microbiol. 2007;45:2144–50. 10.1128/JCM.00174-07 17460053 PMC1933019

[7] Brahic M, Bureau JF, Michiels T. The genetics of the persistent infection and demyelinating disease caused by Theiler’s virus. Annu Rev Microbiol. 2005;59:279–98. 10.1146/annurev.micro.59.030804.121242 16153171

[8] Lipton HL. Human Vilyuisk encephalitis. Rev Med Virol. 2008;18:347–52. 10.1002/rmv.585 18613213

[9] Liang Z, Kumar AS, Jones MS, Knowles NJ, Lipton HL. Phylogenetic analysis of the species *Theilovirus*: emerging murine and human pathogens. J Virol. 2008;82:11545–54. 10.1128/JVI.01160-08 18815294 PMC2583687

[10] Ohsawa K, Watanabe Y, Miyata H, Sato H. Genetic analysis of a Theiler-like virus isolated from rats. Comp Med. 2003;53:191–6. 12784854

[11] Grobler DG, Raath JP, Braack LE, Keet DF, Gerdes GH, Barnard BJ, An outbreak of encephalomyocarditis-virus infection in free-ranging African elephants in the Kruger National Park. Onderstepoort J Vet Res. 1995;62:97–108. 8600443

[12] Reddacliff LA, Kirkland PD, Hartley WJ, Reece RL. Encephalomyocarditis virus infections in an Australian zoo. J Zoo Wildl Med. 1997;28:153–7. 9279403

[13] Drexler JF, Luna LK, Stöcker A, Almeida PS, Ribeiro TC, Petersen N, Circulation of 3 lineages of a novel Saffold cardiovirus in humans. Emerg Infect Dis. 2008;14:1398–405. 10.3201/eid1409.080570 18760006 PMC2603095

[14] Zoll J, Erkens Hulshof S, Lanke K, Verduyn Lunel F, Melchers WJ, Schoondermark-van de Ven E, Saffold virus, a human Theiler’s-like cardiovirus, is ubiquitous and causes infection early in life. PLoS Pathog. 2009;5:e1000416. 10.1371/journal.ppat.1000416 19412527 PMC2670511

[15] Blinkova O, Kapoor A, Victoria J, Jones M, Wolfe N, Naeem A, Cardioviruses are genetically diverse and cause common enteric infections in South Asian children. J Virol. 2009;83:4631–41. 10.1128/JVI.02085-08 19193786 PMC2668475

[16] Abed Y, Boivin G. New Saffold cardioviruses in 3 children, Canada. Emerg Infect Dis. 2008;14:834–6. 18439376 10.3201/eid1405.071675 PMC2600268

[17] Xu ZQ, Cheng WX, Qi HM, Cui SX, Jin Y, Duan ZJ. New Saffold cardiovirus in children, China. Emerg Infect Dis. 2009;15:993–4. 10.3201/eid1506.090109 19523321 PMC2727318

[18] Itagaki T, Abiko C, Ikeda T, Aoki Y, Seto J, Mizuta K, Sequence and phylogenetic analyses of Saffold cardiovirus from children with exudative tonsillitis in Yamagata, Japan. Scand J Infect Dis. 2010;42:950–2. 10.3109/00365548.2010.496791 20608765

[19] Blinkova O, Rosario K, Li L, Kapoor A, Slikas B, Bernardin F, Frequent detection of highly diverse variants of cardiovirus, cosavirus, bocavirus, and circovirus in sewage samples collected in the United States. J Clin Microbiol. 2009;47:3507–13. 10.1128/JCM.01062-09 19794058 PMC2772610

[20] Chiu CY, Greninger AL, Chen EC, Haggerty TD, Parsonnet J, Delwart E, Cultivation and serological characterization of a human Theiler’s-like cardiovirus associated with diarrheal disease. J Virol. 2010;84:4407–14. 10.1128/JVI.02536-09 20164225 PMC2863762

[21] Chiu CY, Greninger AL, Kanada K, Kwok T, Fischer KF, Runckel C, Identification of cardioviruses related to Theiler’s murine encephalomyelitis virus in human infections. Proc Natl Acad Sci U S A. 2008;105:14124–9. 10.1073/pnas.0805968105 18768820 PMC2528868

[22] Ren L, Gonzalez R, Xiao Y, Xu X, Chen L, Vernet G, Saffold cardiovirus in children with acute gastroenteritis, Beijing, China. Emerg Infect Dis. 2009;15:1509–11. 10.3201/eid1509.081531 19788828 PMC2819865

[23] Ren L, Gonzalez R, Xie Z, Xiao Y, Li Y, Liu C, Saffold cardioviruses of 3 lineages in children with respiratory tract infections, Beijing, China. Emerg Infect Dis. 2010;16:1158–61. 10.3201/eid1607.091682 20587195 PMC3321900

[24] Abzug MJ. Perinatal enterovirus infections. In: Rotbart HA, editor. Human enterovirus infections. Washington: ASM Press; 1995. p. 221–38.

